# Persistent hyperlactatemia in decompensated type I diabetes with hepatic glycogenosis and hepatomegaly: Mauriac syndrome: a case report

**DOI:** 10.1186/s13256-022-03416-5

**Published:** 2022-06-02

**Authors:** Waheed Dolip, Eric Bourmanne, Charlotte Van Homwegen, Marc Van Nuffelen

**Affiliations:** Department of Emergency Medicine, Erasme Hospital Belgium, 808 Route de Lennik, 1070 Brussels, Belgium

**Keywords:** Hyperlactatemia, Type I diabetes, Mauriac syndrome, Hepatomegaly, Glycogenosis

## Abstract

**Background:**

We describe a case of Mauriac syndrome, which is a rare complication of poorly controlled type I diabetes that combines glycogenosis, hepatomegaly, growth retardation with a Cushingoid appearance that is most often present in children but also in young adults. Here we also describe another finding with this syndrome, which is hyperlactatemia.

**Case presentation:**

The case is of a 16-year-old female of North African ethnicity with history of poorly controlled type I diabetes who was brought to the emergency department for dyspnea and tachycardia, treated initially for diabetic ketoacidosis. Her persistent hyperlactatemia helped to reveal a more subtle condition known as Mauriac syndrome after multiple examinations and follow-up.

**Conclusions:**

This case reports shows that Mauriac syndrome is a rare condition that should be considered in a setting of poorly controlled type I diabetes, hepatomegaly, Cushingoid appearance, and hyperlactatemia. The current treatment of this condition is a strict control of blood glucose levels with an attempt to achieve an acceptable glycated hemoglobin value.

## Background

Decompensation of diabetes with ketoacidosis is a well-known complication of patients with type 1 diabetes, for which intensive care unit admission is often necessary. Also, in most such cases, lactic acidosis is often present, which is partly explained by impaired glucose metabolism [1, 3].

Lactic acidosis is one of the most common abnormalities found in patients undergoing intensive care and is associated with an increased risk of mortality [3, 5]. Lactic acid is produced from pyruvate via anaerobic respiration in most tissues of the body and especially in the lung and spleen under stress [3]. Metabolism of lactate to glucose occurs mainly in the liver via gluconeogenesis, and this complete cycle of production and conversion to glucose is via the Cori cycle [2, 3]. When lactate production exceeds its clearance, blood hyperlactatemia occurs.

The causes of hyperlactatemia can be divided into two types: A and B. Type A is due to hypoperfusion and tissue hypoxia reflecting a mismatch between oxygen demand and bloodstream supply, resulting in anaerobic glycolysis [2, 3].

On the other hand, type B hyperlactatemia, being more complex, is not associated with any tissue hypoperfusion and can be found in many conditions including liver diseases, neoplastic causes, parenteral nutrition, medication related, mitochondrial diseases, congenital causes, and diabetic ketoacidosis, among others [1–3].

The presence of hyperlactatemia in diabetic ketoacidosis is already known, but it is also often associated with hepatic glycogenosis [1, 4]. Lactic acidosis in diabetic ketoacidosis is usually normalized with correction of blood glucose level and clearance of ketone bodies. This report describes the case of a young woman with persistent lactic acidosis in the setting of poorly controlled type I diabetes, hepatomegaly, and delayed growth with Cushingoid appearance.

## Presentation of the case

A 16-year-old female of North African ethnicity with history of type I diabetes was brought to the emergency department by ambulance for dyspnea and palpitation. She explained that she had a fever of 38.5 °C the day before and that she has had loose stools for a week, with a decrease in her diet, which is why she stopped her insulin treatment. She had a blood pressure of 125/85mmHg, a heart rate of 117 BPM, a respiratory rate of 39/min, a blood sugar level of 655 mg/dl, with ketone bodies present in the urine dipstick. Blood gas analysis showed a pH of 7.09, a lactate level of 2.9 mmol/l (reference value 0.5–2.2 mmol/l) with an anion gap of 44. Initial blood test showed a CRP value of 25 mg/l with hyponatremia of 130 meq/l and hypokalemia of 3.1 meq/l, and moderately elevated liver enzymes. Urine test results did not show any signs of infection, nor did the lung x-ray. Physical examination showed normal lung and heart sound, although there was some tenderness in the abdominal region without any guarding or rebound. The patient was then transferred to the ICU for management of ketoacidosis diabetes.

In the ICU, she was treated conventionally with intravenous hyperhydration, continuous insulin therapy associated with potassium chloride infusion for persistent hypokalemia. Her blood glucose level quickly normalized, and 10% glucose infusion was added in conjunction with continuous insulin therapy until the ketone bodies disappeared, which occurred after 24 hours. However, the blood lactate level continued to rise to 5 mmol/l with the patient in acidemia and compensatory hyperventilation. In view of this, it was decided to increase the dosage of continuous insulin therapy and glucose infusion in parallel, in the hope of forcing metabolization of lactic acid. Unfortunately, this only got worse, and the blood lactate value increased to 12 mmol/l. During this time, the patient’s vital signs remained in adequate ranges, and there was no tachycardia or hypotension. A heart echocardiography was performed, showing increased left ventricular ejection fraction. This ruled out cardiogenic shock and revealed a relative hypovolemia, probably related to dehydration. We thus tried switching to a subcutaneous insulin regimen and continued with intravenous hydration. Unexpectedly, the lactate value started to drop, hyperventilation also decreased, and the blood glucose level stayed around 150 mg/dl.

The clinical examination of the patient during her stay in the ICU department showed a growth delay, significant hepatomegaly, a glycated hemoglobin of 12%, with elevated liver enzymes that persisted with an AST of 180 µ/l, ALT of 82 µ/l, alkaline phosphatase of 135 µ/l, and GGT of 58 µ/l. A hepatic ultrasound examination showed homogeneous hepatomegaly. Given the patient’s very reassuring clinical condition, she was transferred to the endocrinology unit after 3 days in the ICU to pursue her follow-up.

The differential diagnosis for her condition was glycogen overload disease, mitochondrial pathology, hepatopathy in the broadest sense, or an autoimmune polyglandular syndrome. The patient was discharged after 6 days in the endocrinology department, where the dosage for her insulin treatment was adapted. It has also been found that she had family issues and that properly treating herself was not her priority. She was encouraged to see a psychiatrist and was discharged with a basal prandial regimen of three times rapid insulin injection of around 0.25 U/kg and long-acting insulin of around 0.2 U/kg/day. During the 6 months post hospitalization, she continued her follow-up; the results showed a lactate pyruvate ratio in the normal range and a postprandial normal lactate level, making a mitochondrial disease very unlikely. The decreasing lactate level in the fasting state also made the diagnosis of a glycogen overload disease unlikely. Analysis of autoantibodies did not reveal any autoimmune liver disease, and thyroid function was normal. However, the patient continued to show hypertriglyceridemia and hypercholesterolemia. The hyperlactatemia persisted in the patient at 5 mmol/l, causing her to tire very quickly under effort. In this context, an EMG was carried out, showing neither myopathy nor neuropathy. Moreover, on retrieving the patient’s old files, we found the result of a liver biopsy performed 1 year ago in the context of her hepatomegaly for which she had not continued any follow-up. The anatomopathological report showed hepatocytes overloaded with glycogen with some fibrosis. Given this picture of poorly controlled type 1 diabetes, hepatic glycogenosis, growth delay with a Cushingoid appearance, and persistent hyperlactatemia, a diagnosis of Mauriac syndrome was made.

## Discussion

Mauriac syndrome was first described in 1930, in children with type I diabetes treated with rapid-acting insulin, displaying the classical presentation of hepatomegaly with distended abdomen, growth delay, and delay in puberty [6, 7]. Historically, two different forms of this syndrome are described. The first is associated with Cushingoid obesity with fluctuation between documented hypo- and hyperglycemia, resulting in over- and underdosing of insulin treatment [7]. The second form is more recent and occurs in nonobese patients with no history of hypoglycemia or ketoacidosis but who are underdosed with insulin [7]. This syndrome has become rare with the advent of new, long-acting insulin treatments but is often underdiagnosed [8].

The pathophysiology of hepatic glycogenosis in Mauriac syndrome is as follows: After a meal, glucose is transported to the liver and muscles, where it is used for energy production and eventually converted to glycogen, which serves as a storage product for future use. The hepatic glycogen level is a balance between glycogenesis and glycogenolysis. Hyperglycemia causes glucose to enter the hepatocytes via the non-insulin-dependent GLUT2 transporter [9, 10]. Glucose in hepatocytes is irreversibly phosphorylated to glucose 6-phosphate, which is trapped inside the cell [9, 10]. Subsequently, hyperglycemia treated by insulin increases the conversion of glucose 6-phosphate to glycogen by glycogen synthase, which is itself stimulated by insulin and hyperglycemia [9, 10]. Glycogenesis will persist for some time even after the insulin dose has been reduced [10, 11]. Classically, hepatic glycogenosis occurred in patients using excess rapid insulin, which resulted in hypoglycemia, which was corrected by glucose intake, resulting in a vicious circle of hepatic glycogen accumulation [7, 9]. There is also a hypothesis of a genetic defect in the glycogen synthase and glucose 6-phosphatase enzymes, which consequently show overactivity [10, 12]. It is this hepatic glycogenosis that explains the increased level of lactate found in more than half of the population with Mauriac syndrome [4]. This is explained via a reduction of gluconeogenesis via the Cori cycle in the liver, thus increasing blood lactate [4, 5]. There is also a decrease in IGF1 levels and a failure to utilize this glycogen in hepatocytes, which explains the growth retardation in children [7, 9].

In this case, the persistent and initially increasing hyperlactatemia in the ICU despite continuous insulin therapy might have multiple causes including diabetic ketoacidosis and a relative hypovolemic state related to dehydration. This was confirmed as lactate level decreased afterwards with insulin therapy and intravenous hydration.

Although monitoring of blood lactate levels in diabetic ketoacidosis is not routine, this case report shows that this important parameter that can be found easily on blood gas analysis can help to reveal rare cases.

This diagnosis should be suspected in any clinical presentation of a patient with poorly controlled type I diabetes with ketoacidosis, elevated liver enzymes, growth retardation, hepatomegaly, with or without elevated lactate. In terms of imaging, computed tomography (CT) or ultrasound can help differentiate between non alcoholic steatohepatitis (NASH), which is one of the differential diagnoses of hepatomegaly in this type of patient [13]. Magnetic resonance imaging (MRI) is more sensitive for differentiating between steatosis and hepatic glycogenosis, but liver biopsy remains the gold standard where glycogen-loaded hepatocytes are found on Periodic acid–Schiff (PAS) staining [13].

The differential diagnosis of hepatomegaly in type I diabetes with elevated liver enzymes includes NASH, celiac disease, autoimmune hepatitis, viral hepatitis, hemochromatosis, and Wilson's disease. Mitochondrial pathology, which often results in diabetes and hyperlactatemia, must be excluded or the diagnosis of type I diabetes will be misleading [4]. Current treatment consists of strict control of blood glucose levels with attempts to normalize liver enzymes and glycated hemoglobin Hb1c [4, 5, 9].

## Conclusion

In this clinical case, hyperlactatemia was found to be an integral part of the clinical spectrum of hepatic glycogenosis that manifests itself in Mauriac syndrome, which is a rare complication of poorly controlled type I diabetes, found in children as well as adults. Furthermore, this diagnosis should be suspected in any case of long-lasting poorly controlled type I diabetes, accompanied with hepatomegaly, Cushingoid appearance, with hyperlactatemia. The current treatment consists of strict control of blood glucose levels with acceptable glycated hemoglobin values.

## Declarations

### Availability of data and materials

Brugmann hospital, Brussels, Belgium; data available on request.
